# Online Phase Detection Using Wearable Sensors for Walking with a Robotic Prosthesis

**DOI:** 10.3390/s140202776

**Published:** 2014-02-11

**Authors:** Maja Goršič, Roman Kamnik, Luka Ambrožič, Nicola Vitiello, Dirk Lefeber, Guido Pasquini, Marko Munih

**Affiliations:** 1 Faculty of Electrical Engineering, University of Ljubljana, Tržaška 25, Ljubljana 1000, Slovenia; E-Mails: maja.gorsic@robo.fe.uni-lj.si (M.G.); luka.ambrozic@robo.fe.uni-lj.si (L.A.); marko.munih@robo.fe.uni-lj.si (M.M.); 2 The BioRobotics Institute, Scuola Superiore Sant'Anna, viale Rinaldo Piaggio 34, Pontedera 56025, Pisa, Italy; E-Mail: n.vitiello@sssup.it; 3 Vrije Universiteit Brussel, Faculty of Applied Sciences, Pleinlaan 2, Brussels B-1050, Belgium; E-Mail: dlefeber@vub.ac.be; 4 Fondazione don Carlo Gnocchi, Florence 50018, Italy; E-Mail: gupasquini@dongnocchi.it

**Keywords:** wearable sensory system, inertial sensors, instrumented insoles, gait phase detection, robotic prosthesis

## Abstract

This paper presents a gait phase detection algorithm for providing feedback in walking with a robotic prosthesis. The algorithm utilizes the output signals of a wearable wireless sensory system incorporating sensorized shoe insoles and inertial measurement units attached to body segments. The principle of detecting transitions between gait phases is based on heuristic threshold rules, dividing a steady-state walking stride into four phases. For the evaluation of the algorithm, experiments with three amputees, walking with the robotic prosthesis and wearable sensors, were performed. Results show a high rate of successful detection for all four phases (the average success rate across all subjects >90%). A comparison of the proposed method to an off-line trained algorithm using hidden Markov models reveals a similar performance achieved without the need for learning dataset acquisition and previous model training.

## 1. Introduction

Prostheses efficiently replace a lost limb after amputation. Lower-limb prostheses enable the recovery of functional movements in the everyday life of an amputee, such as standing up, walking and stair climbing. Technically, lower-limb prostheses have evolved from simple passive walking aids, attached to a stump, to complex devices that incorporate damping mechanisms, microprocessor control and also actuators, together aiming at achieving symmetrical, stable and more energy-efficient motion.

The state-of-the-art of commercially available prostheses for lower extremities incorporates advanced design based on lightweight materials and passive components for assuring more human-like walking. Commercial products enable walking at ground-level at different speeds, while only some of them are appropriate for slope and stairs negotiation. Users report many substantial problems with prostheses usage reducing their quality of life [1,2]. Walking at a higher speed is asymmetrical [3], while the energy expenditure increases [4]. For performing step-over-step stair climbing, slope ascending or standing-up from a seated position, the prosthesis should be capable of contributing to the lifting forces. However, powered prostheses are still rare on the market, and they are lacking a conceptual control based on human activity observation.

Several robotic-driven lower-limb prostheses have been developed as research prototypes. Different configurations employ fully active, or semi-active only knee- [5–8], only ankle-foot- [9–11] or combined knee-ankle- [12–14] driven joints. Active prostheses are controlled by impedance [6], finite-state [5,7,8,13,14], echo [15] or myoelectric control [10]. Feedback information is typically provided to the controller using integrated encoders, sensors measuring the interaction forces, EMG (electromyography) electrodes or inertial sensors. Common to known approaches is that the robotic driven joints are operating on the basis of locally assessed information about the current state in the gait cycle. Sensors are attached directly on the prosthesis or near the interaction points. With local assessment, the information about human motion dynamics is incomplete. The EU FP7project, CYBERLEGs (The CYBERnetic LowEr-Limb CoGnitive Ortho-prosthesis), aims at the development of an active robotic prosthesis for above-knee amputation, controlled by a whole body wearable sensory system. Whole body awareness promises more precise assessment of the current motion status, thus enabling richer feedback information and better closed-loop control.

Detection of walking phases is a basic prerequisite for controlling the prosthesis in finite-state mode. For providing feedback information on the subject's activity and, at the same time, not restricting tge subject's movements in everyday life, the sensors must be wearable and wirelessly connected to an acquisition unit. Feet interaction force sensors and inertial sensors have proven to be adequate and reliable for assessing kinetic and kinematic human motion parameters.

Known rule-based phase detection algorithms [16,17] are based on the detection of peaks in acquired signals, which complicates the usage in online real-time operation. On the other hand, machine learning methods [18–23] require the acquisition of data and model training in advance, thus posing an inconvenient requirement for clinical and home environments. Phase detection algorithms running in real time have been developed; however, only a few specific events of a walking stride, like initial contact and heel off, were detected [16,18,24,25]. Real-time threshold-based detection of initial contact using wearable sensors was experimentally evaluated by Hanlon *et al.* [18] on 12 healthy subjects. The performance of a threshold-based algorithm using footswitch sensors turned out to be more accurate than local peak detection in signals acquired from an accelerometer sensor. With gyroscope usage, over a 98% success rate was achieved for the detection of initial contact and foot off events by Catalfamo *et al.* [24], evaluated on seven healthy subjects. Algorithms based on hidden Markov models are a common approach for gait segmentation [19,20]. With wearable shoe insoles, a high average success rate (96%) for walking phase detection was achieved by Crea *et al.* [23] using hidden Markov models (HMMs) evaluated on five healthy subjects. With a multisensory shoe system, Bamberg *et al.* [25] detected the heel strike and toe off timing accurately during healthy and pathological gait. With the implementation of accelerometers, gyroscopes, force sensors, dynamic pressure sensors, bidirectional bend sensors and electric field height sensors on subjects' walking shoes, they were able to estimate the foot orientation and position without interfering with human motion. Papas *et al.* [26–28] developed a simple phase detection algorithm, dividing the walking stride into four parts: heel off, swing, heel strike and stance phases. Three force sensitive resistors and a gyroscope were implemented in the shoe, not taking into account the kinematics of other body segments. Evaluation experiments in combination with functional electrical stimulation outlined the lack of wireless communication between sensors and the controller as inconvenient.

This paper presents an algorithm for real-time stride cycle phase detection utilizing a whole body wireless wearable sensory system. In the methodology, the algorithm is formulated as a state machine with threshold-based transition rules. The experimental evaluation involving amputees walking with a robotic prosthesis is described. Phase detection performance is compared to an alternative approach using hidden Markov models. The main objective of the paper is to present a proof of concept for closing the loop with humans and the robot and sensory technology currently being developed in a real scenario.

## 2. Methodology

### 2.1. Walking Phases

**Figure 1.**
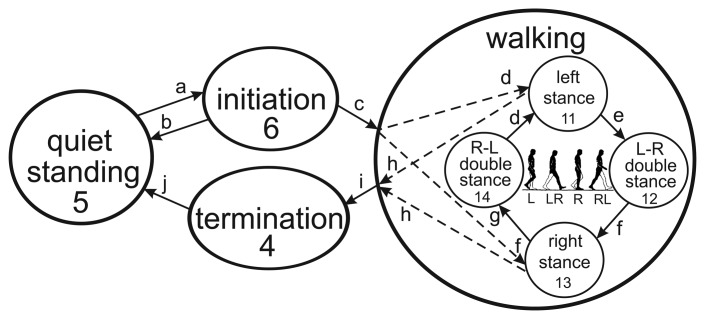
The state diagram of the intention detection algorithm and prosthetic control.

Gait is the most common and also one of the most complex human activities [29,30]. The ground-level walking cycle is presented in Figure 1. Quiet standing is a starting pose for bipedal locomotion. The walking maneuver begins with gait initiation. Gait can be initiated by either the left or the right leg, followed by steady-state cycles of steps. During a steady-state gait, the single and double stance phases alternate until gait termination occurs, followed by quiet standing. The single stance period occurs when only one leg is in contact with the ground, while the double stance period is present when this is valid for both legs. Left to right (L-R) double stance occurs after the left single stance phase, and right to left (R-L) double stance occurs after the right single stance phase. Phase durations vary with the speed of walking: the faster the walking, the shorter the double stance phases are.

Control of the robotic prosthesis requires the identification of particular phases in real time. The state diagram for prosthesis control consists of four main states, with the walking maneuver further divided into four phases. In Figure 1, the states are marked with numbers and the transitions with letters.

### 2.2. Wearable Sensors

**Figure 2.**
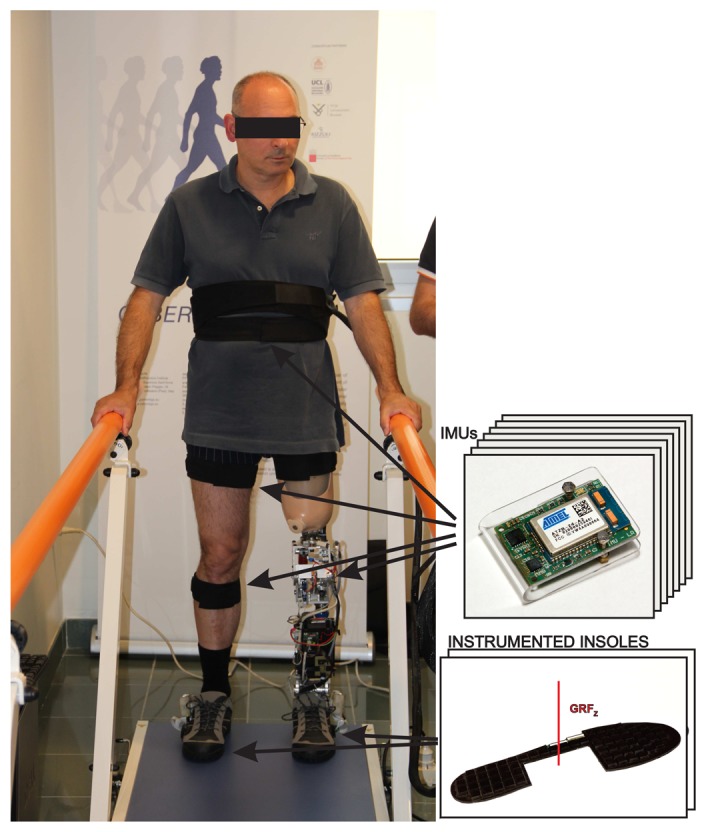
The experimental setup: the amputee is walking between parallel bars with a robotic prosthesis and wearing wearable sensors.

The wearable sensory system for providing input information to the phase detection algorithm consists of two wireless pressure-sensitive shoe insoles and seven inertial measurement units (IMUs) attached to human body segments, as presented in Figure 2.

The instrumented shoe insoles, developed at Scuola Superiore Sant'Anna, Pisa, Italy [31–33], consist of 64 pressure cells, each with electronics for signal conditioning and wireless data transmission. Each cell is made of a silicone-covered opto-electronic pressure sensor, which outputs the electrical signal with regard to the occlusion of the light path. An unamplified analog voltage signal is acquired by on-board electronics and is proportional to the deformation on the cell, due to the applied load. The instrumented insole can replace a regular insole in normal sneaker shoes of EU size 42-44 and does not interfere with the normal gait pattern. A receiver unit acquires data from two insoles via the Bluetooth communication protocol. Insoles are powered by an on-board battery and output computed feet reaction forces and load distribution expressed as the center of pressure.

Inertial measurement units (IMUs) have been developed to measure the orientation of body segments [34,35]. The units are small (30 × 20 × 5 mm), lightweight (6 g) and can be attached to body segments unobtrusively. IMU-based measurement of kinematic parameters is not restricted to a certain measurement field and can be used to collect data on the movements of multiple segments. The inertial and magnetic measurement system consists of 7 inertial measurement units. Each IMU contains miniature MEMS (micro-electromechanical systems) sensors, including a 3D accelerometer (range: ±2 g), a 3D gyroscope (range: ±500°/s) and a 3D magnetometer (range: ±1.3 G), as well as an on-board 8-bit processor. All sensors are connected to an inter-integrated circuit (I2C) bus with a maximum data transfer rate of 222 kb/s. The IMUs utilize the wireless transmission of the data packages to the main acquisition unit for fetching sensory data, which consists of measured vectors of acceleration, angular velocity and the heading of the magnetic field. IMUs are calibrated after assembly for axis misalignment and before experimental trials for determining the magnetometer's bias, misalignment and gain.

For assessing human motion kinetics and kinematics, a multitude of sensors were used. One insole was placed under the sound leg and the other under the prosthesis. Six IMUs were attached on leg segments (thighs, shanks, feet) using soft, elastic straps with silicone lining to prevent slipping. One IMU was placed near the lumbosacral joint on the back. Raw signals were collected at 100 Hz by the main acquisition unit and fused by an unscented Kalman filter to determine segment orientations [36,37].

### 2.3. Initial Dataset Acquisition

In order to acquire an initial dataset from the sensory system and to gain knowledge on walking characteristics, preliminary tests on healthy subjects were performed. Five healthy subjects (27.7 ± 5.0 years old, 171.3 ± 5.2 cm in height, 70.8 ± 3.5 kg in weight) were instrumented with the wearable sensory system and instructed to walk along a 10 m-long straight pathway at their preferable speed. From the acquired dataset of raw sensory signals, the most descriptive outputs regarding phase transitions were chosen for inclusion in the algorithm. The selected sensory signals and their derived variables are listed in Table 1. Walking phases were manually identified, referring to characteristic transitions of the chosen signals. We intentionally excluded walking trials with amputees walking with their own passive prosthesis, since the walking pattern incorporates various compensating moves.

The signals that describe the feet interaction with the ground are the ground reaction forces of the left (grfL) and right (grfR) foot. The foot load distributions along the longitudinal axis are the position of the center of pressure of the left (COPyL) and the right (COPyR) foot. The average absolute difference between both ground reaction forces of the feet for the past 50 samples, describing the temporal lateral asymmetry, is denoted as grfDiff. Raw signals from the IMU gyroscope on the feet represent the angular velocity of the left (gyroL) and the right (gyroR) foot in the sagittal plane. Kalman filter output signals, describing hip and knee joint angles, were summed into a single signal, labeled as sumAng. The signal corresponds to the amount of flexion of the lower extremities.

### 2.4. Transition and Walking Phase Detection Algorithm

Signals, listed in Table 1, represent an input dataset for the transition and phase detection algorithm. On the identified signals, the thresholds were defined, determining the transitions between states. The thresholds are collected in Table 2, denoted by the labeled tags. Threshold QSgrf is the value of the ground reaction force delimiting quiet standing and the motion state. stanceL and stanceR are thresholds defined also for ground reaction force signals detecting whether the foot is in contact with the ground or not. Two thresholds are specified for the grfDiff signal to determine transitions between quiet standing and initiation or termination (init1) and between initiation or termination and the walking maneuver (init2). Another two thresholds were specified for the center of pressure signals, differentiating between L-R and R-L double stance phases (midCOP, toeCOP). For the sumAng signal, three different thresholds were defined for discriminating between quiet standing and initiation (sumAngInit), quiet standing and termination (sumAngTerm) and double stance states during the walking maneuver (minAng). For foot angular velocity signals (gyroL, gyroR), two thresholds were defined, one for detecting the minimal movement of the foot, denoting the transition to the initiation state (minG) and one for detecting the transition to the walking state (initG). All threshold values were accessible via a specially designed graphical user interface (GUI) for fine-tuning.

Based on a thorough analysis of the evident transitions in the signals, the combinations of rules for transition and phase detection were defined (Table 3). For detecting the starting position for quiet standing, the following conditions must be met. In an upright body posture, the legs should be extended (sumAng < sumQS), and the ground reaction force must be symmetrically distributed between both feet (grfDiff < init2). From the termination to the quiet standing state (see Figure 1, Table 3, transition j), the angular velocities on the feet must be low enough to ensure that both feet are standing still ((gyroL < minG) AND (gyroR < minG)). The initiation state (transition a) starts with the initial flexion of the starting foot (sumAng > sumAnglnit) along with the initial lateral asymmetry in ground reactions ((grfDiff > init1) AND ((grfL < QSgrf) OR (grfL < QSgrf))). For detecting the termination state (transitions h and i), the opposite situation is required. The body should be near the upright posture (sumAng < sumAngTerm), both feet must be in contact with the ground ((grfL > QSgrf) AND (grfL > QSgrf)), ground reactions should be symmetrical (grfDiff < init1) and the feet should be standing still ((gyroL < termG) AND (gyroR < termG)).

For steady-state walking, the detection conditions for four phases are determined. The left stance (transitions c and d) occurs when the left foot is in contact with the ground (grfL > stanceL), while the right foot is up in the air (grfR < stanceR), flexion in the legs is detected (sumAng > sumAnglnit) and the lateral asymmetry of the reaction forces is high (grfDiff > init1). For the right stance state (transitions c and f), the conditions are the same, except that the right foot is in contact with the ground (grfR > stanceR) and the left foot is in the air (grfL < stanceL). Double stance phases are detected after a single stance, when both feet are touching the ground ((grfL > stanceL) AND (grfR > stanceR)), with some flexion in the legs remaining (sumAng > minAng). To distinguish between the L-R and the R-L double stance, the load distribution of the ground reaction force is examined. For the L-R double stance (transition e), the reaction under the left foot acts primarily under the toes (COPyL < midCOP), while the reaction under the right foot acts primarily on the heel (COPyR > toeCOP). For the R-L double stance phase (transition f), the conditions are the opposite ((COPyL > toeCOP) AND (COPyR < midCOP)). All the conditions collected in Table 3 form the basis for an expert knowledge rule-based algorithm for transition and phase detection in walking.

For performance comparison, an alternative algorithm encompassing hidden Markov models (HMMs) was built using supervised learning. The hidden Markov model is a statistical model in which the modeled system is assumed to be a Markov process with unobservable states and observable outputs (observations are, in our case, the measured signals). The HMM only characterizes the statistical properties of the system. The model is defined by the number of states (the more states, the more complex the model), the number of outputs, the state transition probabilities, the output probabilities and the initial state probability distribution. For each gait phase, a three-state HMM was trained, using the same combination of input signals as in the previously described rule-based algorithm. The learning set was constructed as a set of signal patterns defining particular walking phases. The technique of maximum likelihood estimation was used to train the model's state probabilities and output parameters. For HMM construction, a free online available toolbox developed by Kevin Murphy was used [38]. Phase recognition was evaluated using a window of the current and past 9 samples of the input data. The likelihood for each phase model was computed, and the maximum of all four likelihoods determined the walking phase that belonged.

### 2.5. Experimental Evaluation

#### 2.5.1. Subjects

Three persons following trans-femoral amputation (loss of lower limb) were recruited for testing the alpha prototype of the CYBERLEGs prosthesis with closed-loop control and feedback from the proposed motor intention recognition. All subjects provided consent with involvement in the study, and the experiments were approved by the governing ethical committee within the research scope of the project. The criteria for the selection of subjects were that these were persons following a transfemoral amputation (for any reason) and were of good general health. There were difficulties with recruiting amputees that were capable and willing to participate in the experiments. According to studies from the literature, a small sample size of test subjects is sufficient to prove the conceptual functionality of the tested system [39]. The subject characteristics are presented in Table 4.

The subjects (59.7 ± 11.0 years old, 173.3 ± 5.8 cm in height, 60.5 ± 2.65 kg in weight) have had a traumatic amputation, and all three wore the prosthesis with an ISNY socket.

#### 2.5.2. Measurement Setup

The measurement environment consisted of a walkway with handrails for directing and assuring safety to the testing subject during the experimental walking trials. The testing subjects wore the CYBERLEGs alpha-prototype robotic prosthesis [40] and wearable sensory system, encompassing two wireless insoles and seven inertial measurement units together with the receiver units. Figure 2 shows an amputee in the measurement environment wearing the robotic prosthesis and sensory system.

The prosthesis incorporates a full torque-enabled active ankle and a passive knee mechanism. The knee is a spring-loaded mechanism with some pretension, and it stores the negative knee work in a passive element during walking. It is able to reproduce the knee torque-angle requirements in steady-state walking via a locking mechanism, thus approximating normal knee behavior. The ankle is driven by a MACCEPA (The Mechanically Adjustable Compliance and Controllable Equilibrium Position Actuator) compliance actuator, which is designed according to the benchmark criteria of an 80-kg individual that tends to walk at a normal speed of 1 stride per second. Variable stiffness assures the achievement of the desired stiffening characteristic.

Sensory signals were acquired via a wireless transmission by two acquisition units and were processed by a desktop PC running in real time (Mathworks xPC Target). Another PC computer was used as a host computer for control scheme development, debugging and data fetching.

The prosthesis was controlled by a National Instruments CompactRIO (Reconfigurable I/O) system based on a real-time processor with an FPGA (field-programmable gate array) layer, operating as a finite-state machine controller. The input to the state machine was the phase identifier from the phase detection algorithm. The controller changed the resistive torque in the knee joint to follow predefined patterns, synchronized with the stance and swing phase of the gait. Sensory signals fed to the controller incorporate joint positions, torque estimations and insole loading data. The control concept was implemented as a rule-based system with the transitions following the identified gait state, controlling the knee locking, ankle angle and ankle torque.

#### 2.5.3. Experimental Protocol

**Figure 3.**
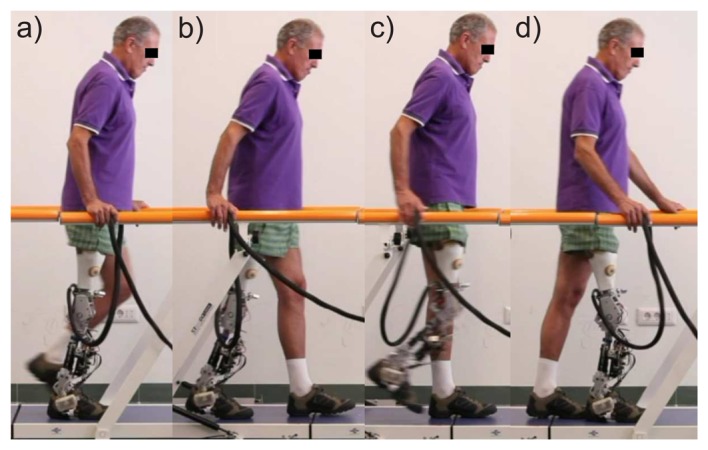
Steady-state gait phases of an amputee walking. From left to right: (**a**) single stance prosthetic limb (*SS_p_*); (**b**) double stance prosthetic-sound limb (*DS_ps_*); (**c**) single stance sound limb (*SS_s_*); and (**d**) double stance sound prosthetic limb (*DS_sp_*).

The experimental protocol required amputees to perform 6 meter-long walks with the closed-loop controlled prosthesis at their preferred speed and step length. The time for experiments was limited for each participant. The protocol was planned in a way that the subject could familiarize himself with the prosthesis and perform from five to ten test walks prior to data recording. This was also an opportunity for the investigators to fine-tune the subject-specific parameters, based on weight, height and the side of the prosthetic leg. In Figure 3, the walking phases for a single stride cycle of an amputee are presented. For comparison among amputees, the phases were determined according to the side of the sound and prosthetic leg instead of left and right side. Denotation *SS_s_* stands for the single stance phase of a sound leg and *SS_p_* for the single stance of a prosthetic leg. *DS_sp_* denotes the double stance phase with the sound leg in the back and the prosthetic leg in the front, while in the double stance denoted by *DS_ps_*, the legs are in the opposite position.

The phase detection algorithm phases were a direct input for the allowance of transitions in the finite-state control mechanism of the prosthesis. The finite-state controller allowed only for a full-gait cycle to be performed or for a termination state (if stopping). If the detected phase was in accordance with the gait cycle, the control mechanism triggered a state transition; otherwise, it remained in the given state (control was paused) until the input triggered an allowed transition. However, the phase detection algorithm was not restricted to the gait cycle series; therefore, it allowed for a gait phase to be skipped if not detected, until the next was detected correctly. Once the previously undetected phase was detected, control of the prosthesis resumed. During paused control, the prosthesis behaved as a passive support that the amputee had to pull up and bring forward. If the passive transition was successful, the trial continued; otherwise, the trial was aborted and a new trial conducted. Phases of the whole gait cycle were noted as not detected.

When sensors are attached to the human body, the subject is required to stand still for at least five seconds for the initial calculation of the segments' orientation. The goal was to perform 25 walking trials with each subject. The first amputee, *S1* (subject 1), finished only 15 trials, due to problems with the prosthesis operation on the first day. Subjects *S2* and *S3* accomplished 25 walks each. Subject *S3* was available for experiments for two consecutive days and thus completed an additional 23 walks.

#### 2.5.4. Data Processing

With a combination of expert knowledge and supervised automated checking of the phase sequence, the acquired sensory data of the steady-state gait were segmented into single and double stance phases. The acquired data from all walking trials represented the reference dataset for verification of the transition and phase detection algorithm. The correctness of the identified phase was verified by checking the correct phase sequence pattern. The accuracy was evaluated as the success ratio between the number of correctly recognized phases and the number of all phases of a particular type.

For the alternative approach using HMM, three different sets of data were used to train the models. First, individual HMMs were trained for each subject using individual data, demonstrating the intra-subject phase detection performance. Second, models were trained on data encompassing sets of two subjects excluding the verified one, testing the inter-subject accuracy of phase detection. Third, data from all three amputees together was used to train an HMM, verifying the generalization capability of the HMM approach. All data analysis was performed using MATLAB software.

## 3. Results and Discussion

The presented results demonstrate the performance of the rule-based algorithm for the detection of walking phases. The number of recorded walking trials for each subject is presented in Table 5. Within the measured walks, subjects made a certain number of steps via a certain number of walking phases.

The subject IDs are listed in the first column. The number of recorded walks and corresponding gait phases accomplished by each subject are presented in the second and third column, respectively. The last two columns describe the amount of data used for training and for evaluating the alternative algorithm with HMM. The number of datasets used for training HMMs was intentionally left small, mimicking a real usage situation in which the acquisition of a large dataset prior to usage is not practical.

**Figure 4.**
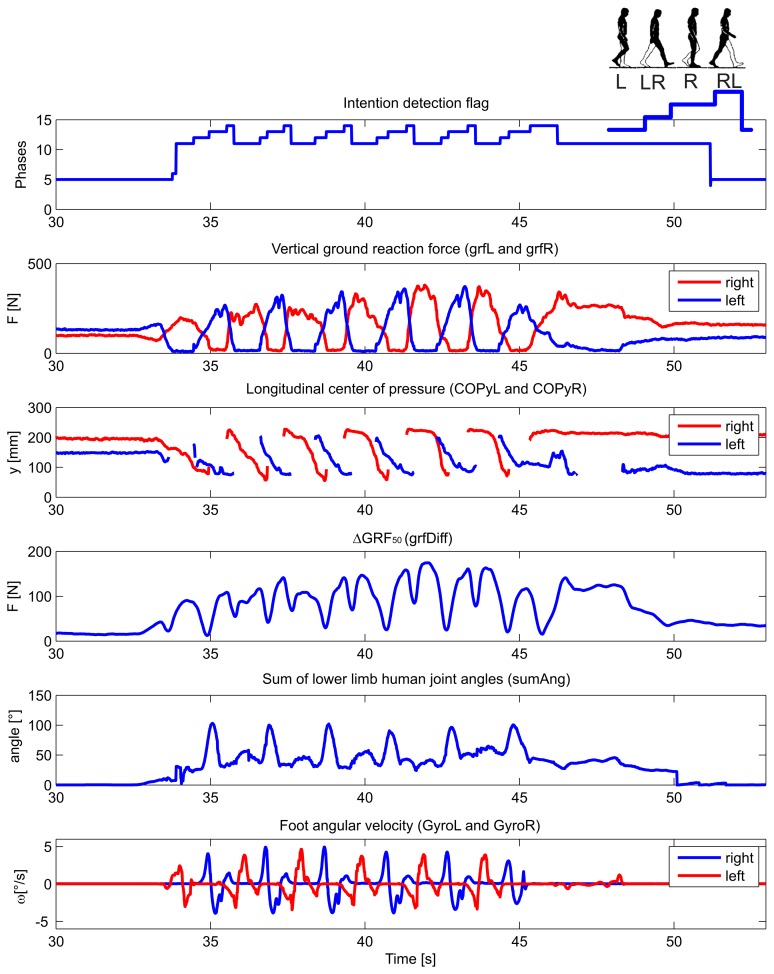
Typical selected input signals and output of the algorithm for a subject during a walking trial. In the top right corner, the pattern sequence for a single stride is illustrated with L for left stance, L-R for left-right double stance, R for right single stance and R-L for right-left double stance. The phase flag values correspond to the values 11, 12, 13 and 14, defined in Table 3.

The detection of walking initiation and termination by the wearable sensory system was thoroughly evaluated elsewhere [41]. Typical wearable sensory signals and their derivatives (listed in Table 1) acquired during the experimental walking trial of amputee *S2* are shown in Figure 4. The numerical output of the transition and phase detection rule-based algorithm is shown in the top-most graph, presenting the corresponding values from Table 3. The graph denoted as vertical ground reaction force shows the loading of the sensorized insoles, for the right and left foot, separately The graph below presents the longitudinal position of the center of pressure under the right and left foot. The graph marked as Δ*GRF*_50_ plots the average absolute difference between the left and right vertical ground reaction force over a window of the past 50 samples. The second graph from the bottom shows the sum of lower limb joint angles (hips and knees), while the bottom-most graph shows the angular velocity of the foot in the ankle's sagittal plane. All signals are plotted with respect to time. In the top right corner, the pattern sequence for a single stride is illustrated with L for left stance, L-R for left-right double stance, R for right single stance and R-L for right-left double stance. The output flag values correspond to values 11, 12, 13 and 14, as defined in Table 3.

In Table 6, the performance of the rule-based phase detection algorithm is presented. The detection success ratios are listed in columns for all four walking phases, with denotations corresponding to Figure 3. In the last column, mean values over all phases are presented.

The results demonstrate that the achieved overall success rate for all four subjects is over 90%. The highest achieved average success rate over all subjects is with detection of sound leg single stance phase *SS_s_*, which implies that the loading pattern and kinematic parameters during sound leg usage are similar to those in healthy subjects. The lowest success rate is outlined for detection of sound-prosthetic double stance phase *DS_sp_* in which the body weight transfer from the sound to the prosthetic side commences. In this transfer, with the new prosthesis, amputees had to adopt a new pattern. Adaptation can be observed for subject *S3*, who participated in measurement trials through two consecutive days. Success ratios improve from day 1 (*D1*) to day 2 (*D2*) for all phases which involve the prosthetic leg (*SS_p_*, *DS_sp_*, and *DS_ps_*) implying higher confidence in walking with the prosthesis.

Table 7 shows results for phase detection using an alternative approach with HMMs. First section presents the results of intra-subject verification of HMM performance for each particular subject, second section shows the inter-subject HMM verification results and the last section results for generalized HMM usage. Training sets used for verification are listed in the first row and the set construction is denoted with corresponding subject IDs, where Si denotes ID of currently evaluated subject.

For all three variations, the best results for gait phase detection are evident for subject *S1*, who was the only one familiar with the prosthesis prior experiments. Due to increased confidence in usage of the prosthesis a repeatable walking pattern is recognized with high success rate of phase detection (>90%). Walks of the other two subjects were more insecure with a less consistent pattern, while they also used trail handles to maintain support and balance. The best performance among the three HMM options, between 89% and 99%, is demonstrated by the generalized model, *S1* + *S2* + *S3*, as it was trained over the richest amount of training data.

The lowest success rate can be observed in detection of *DS_ps_* phase in intra-subject HMM verification for subject *S3* during both days. As this subject had a recent amputation (3 years ago) and had problems walking even with his own prosthesis, the transfer phase *DS_ps_* was difficult to accomplish for him. From the results it can be concluded that his transfer pattern was atypical, since success ratios for intra-subject verification are high (83.3% and 98.0%), drop for generalized model (76.7% and 88.2%) and are low for inter-subject verification (15.8% and 25.5%). Also, the lowest success ratio for *DS_ps_* phase is observed in generalized model and intra-subject verification for all subjects.

Comparing the results of both approaches, the performances of generalized HMM and rule-based phase detection algorithm are similar. Other two HMMs configurations performed less accurately in at least one phase recognition. For the generalized model and inter-subject verification the lowest success ratio is evident for *DS_ps_* phase, similar to the rule-based algorithm. Also, performance improvement is demonstrated for both approaches in case of adaptation of subject *S3*, i.e. success ratios for phases *SS_p_*, *DS_sp_* and *DS_ps_* improve from day 1 to day 2 for *S3* intra-subject validation.

The presented performances are affected by disadvantages of wearable sensors usage. Signals from the sensory system are noisy in nature, while additional filtering imposes delay in phase detection. Sensors must be placed on the body segments in a way that its axes are aligned with segments' sagittal plane. In practice, this alignment is approximate. For exact alignment an additional calibration procedure would need to be accomplished after sensors' placement. Due to muscle contractions and extensions, the transformation between the segment and the sensor axes constantly changes within a limited range. The deterioration was comprehended by tuning the initial threshold parameters of the algorithm for each subject prior to use. As the main limitation of our study we recognize the evaluation with a small number of testing subjects, as this presented a compromise between complexity of the experiment and the size of testing set. Namely, the study focuses on proving the concept of a simple real-time phase detection method in a real case scenario with three amputee subjects. However, a follow-up clinical study is planned in the future, incorporating improved sensory hardware and recognition in additional tasks of human locomotion in order to statistically describe the system performance within larger subject set.

## 4. Conclusions

We developed a rule-based gait phase detection algorithm based on signals from the wearable wireless sensory system. The algorithm was evaluated for walking of three amputees wearing a robotic prosthesis controlled by a finite-state controller, in which the state transitions and the joint trajectory generations were driven by phase detection. The proposed approach was compared to an alternative algorithm using HMMs.

The outlined results demonstrate that the wearable phase detection in walking with the robotic prosthesis can be performed with average success rate across all subjects higher then 90% for all phases and that its employment in closed loop control of the prosthesis is functional. The proposed algorithm is computationally simple and consists of 18 heuristic rules for checking, which enables the implementation on a wearable microcontroller and real-time operation. The achieved performances in phase detection of the rule-based algorithm are comparable to the alternative HMM approach. Main concept is proven to be useful in real scenario, although evaluation on larger sample size is needed in the future. We also plan to expand the algorithm for other motion maneuvers such as stair climbing and stand-to-sit maneuver.

The presented algorithm embodies a white box approach enabling easy implementation. A simple structure with evidently defined rules and tunable thresholds allows quick adaptation to the user. The major advantage over machine learning methods is that the measurement walking trials for data acquisition and manual pattern segmentation prior usage are not needed.

## Figures and Tables

**Table 1.** Chosen input signals for the transition and phase detection algorithm. The left column lists signal tags and the right column the corresponding descriptions.

**Input Signals**	**Explanation**
grfL	vertical ground reaction force of the left foot (N)
grfR	vertical ground reaction force of the right foot (N)
grfDiff	average absolute difference between grfL and grfR in the past 50 samples (N)
COPyL	longitudinal coordinate of the center of pressure for the left foot (starting from the toes to the heel) (mm)
COPyR	longitudinal coordinate of the center of pressure for the right foot (starting from the toes to the heel) (mm)
sumAng	the sum of all knee and hip angles (°)
gyroL	angular velocity of the left foot in the sagittal plane (rad/s)
gyroR	angular velocity of the right foot in the sagittal plane (rad/s)

**Table 2.** Thresholds defined for the input signals from Table 1. The left column consists of threshold tags and the right column the corresponding descriptions.

**Thresholds on Input Signals**	**Explanation**
QSgrf	threshold for grfL and grfR signals determining the quiet standing state
stanceL	threshold for the grfL signal determining the left stance phase
stanceR	threshold for the grfR signal determining the right stance phase
sumQS	threshold for the sumAng signal determining the quiet standing state
init1	threshold for the grfDiff signal determining the initiation state or the termination state
init2	threshold for the grfDiff signal determining the walking state
midCOP	threshold for the COPyL and COPyR signals, determining the double support phases during the walking state
toeCOP	threshold for the COPyL and COPyR signals, determining the double support phases during the walking state
sumAnglnit	threshold for the sumAng signal determining the initiation state
sumAngTerm	threshold for the sumAng signal determining the termination state
minAng	threshold for the sumAng signal determining the double support phase in the walking state
minG	threshold for the gyroL and gyroR signals determining the minimal movement of the feet used for the quiet standing state
termG	threshold for the gyroL and gyroR signals determining the termination state of the feet used for the transition to walking

**Table 3.** The defined conditions for state machine transitions. The left-most column describes the recognizable states, the second column the specific rule combinations for achieving transitions (see Tables 1 and 2), the third column the flag number describing the state (as in Figure 1) and the last column the transition designator, corresponding to the transitions presented in Figure 1.

**State**	**Condition**	**Flag Number**	**Transition Designator**
Quiet standing from initiation	(grfDiff < init2) && (sumAng < sumQS)	5	b

Quiet standing from termination	(grfDiff < init2) && (sumAng < sumQS) && && (abs(gyroL) < minG) && (abs(gyroR) < minG)	5	j

Initiation	(grfDiff > init1) && ((grfL < QSgrf) ‖ (grfR < QSgrf)) && && (sumAng>sumAngInit)	6	a

Termination	(grfDiff < init1) && (grfL > QSgrf) && && (grfR > QSgrf) && (sumAng < sumAngTerm) && && (abs(gyroL) < termG) && (abs(gyroR) < termG)	4	h & i


Walking			

Left stance	(grfDiff > init1) && (sumAng > sumAnglnit) && && (grfL > stanceL) && (grfR < stanceR)	11	c & d

Left-right double stance	(grfR > stanceR) && (grfL > stanceL) && (COPyL < midCOP) && && (COPyR > toeCOP) && (sumAng > minAng)	12	e

Right stance	(grfDiff > init1) && (sumAng > sumAnglnit) && && (grfL < stanceL) && (grfR > stanceR)	13	c & f

Right-left double stance	(grfR > stanceR) && (grfL > stanceL) && (COPyL > toeCOP) && && (COPyR < midCOP) && (sumAng < midAng)	14	g

**Table 4.** Test subjects.

**Subject**	**Sex**	**Age**	**Weight**	**Height**	**Amputated Limb**	**Socket Type**	**Current Prosthesis**	**Year of Amputation**
S1	M	66	58.5	180	Right	ISNY	C-Leg(Otto Bock)	2003
S2	M	47	63.5	170	Left	ISNY	Monocentric knee with hydraulic friction	1982
S3	M	66	59.5	170	Right	ISNY	Nabtesco polycentric knee	2010

Weight without prosthesis; ISNY - Icelandic-Swedish-New York above-knee prosthetic sockets.

**Table 5.** Number of walks, acquired gait phases and hidden Markov model (HMM) dataset configuration for each subject (S1, subject 1; S2, subject 2; S3 D1, subject 3, day 1; S3 D2, subject 3, day 2).

**Subject ID**	**Number of Walks**	**Number of all Measured Gait Phases**				**Number of Walks for Training HMM**	**Number of Walks for Evaluating HMM**

		***SS_s_***	***SS_p_***	***DS_sp_***	***DS_ps_***		
S1	15	91	78	78	77	3	12
S2	25	128	115	107	110	3	22
S3D1	23	129	124	113	116	0	23
S3D2	25	132	133	117	131	3	22

**Table 6.** Success rates for the online detection of gait phases using the rule-based algorithm.

**Subject ID**	***SS_s_*** **(%)**	***SS_p_*** **(%)**	***DS_sp_*** **(%)**	***DS_ps_*** **(%)**	**Mean** **(%)**
S1	92.3	96.2	96.2	96.1	95.2
S2	100	99.1	95.3	99.1	98.4
S3D1	99.2	99.2	85.8	97.4	95.4
S3D2	99.2	100	88.9	98.5	96.7

All Subjects	99.7	98.6	91.6	97.8	96.9

**Table 7.** Success rates for detection of gait phases using hidden Markov models.

**Training Set**	**Si [%]**				**S1 + S2 + S3-Si [%]**				**S1 + S2 + S3 [%]**			
			
Subject ID	*SS_s_*	*SS_p_*	*DS_sp_*	*DS_ps_*	*SS_s_*	*SS_p_*	*DS_sp_*	*DS_ps_*	*SS_s_*	*SS_p_*	*DS_sp_*	*DS_ps_*
S1	93.2	100	100	100	95.9	100	93.6	95.1	94.5	100	100	100
S2	80.6	97.2	100	75.8	98.1	100	95.4	51.6	100	96.8	98.2	94.7
S3D1	82.4	96.2	83.3	89.1	96.2	97.0	15.8	99.2	95.4	100	76.7	98.3
S3D2	53.8	100	98.0	99.0	81.1	98.3	25.5	99.0	88.7	99.1	88.2	98.1
			
All Subjects	76.5	98.1	94.2	90.2	92.7	98.5	52.7	86.5	94.7	99.0	89.3	97.6
